# Validation of an advanced practice physiotherapy model of care in an orthopaedic outpatient clinic

**DOI:** 10.1186/1471-2474-14-162

**Published:** 2013-05-08

**Authors:** François Desmeules, Panagiota Toliopoulos, Jean-Sébastien Roy, Linda June Woodhouse, Marc Lacelle, Manon Leroux, Steven Girard, Debbie E Feldman, Julio C Fernandes

**Affiliations:** 1 School of Rehabilitation, Faculty of Medicine, University of Montreal, Montreal, QC, Canada; 2 Orthopaedic clinical research unit, Maisonneuve-Rosemont Hospital Research Center, University of Montreal Affiliated Research Center, Montreal, QC, Canada; 3 Department of Rehabilitation, Faculty of Medicine, Laval University, Quebec City, QC, Canada; 4 Centre for Interdisciplinary Research in Rehabilitation and Social Integration, Quebec City, QC, Canada; 5 Department of Physical Therapy, Faculty of Rehabilitation Medicine, University of Alberta, Edmonton, AB, Canada; 6 McCaig Institute for Bone and Joint Health, Calgary, AB, Canada; 7 Department of Physiotherapy, Sacré-Coeur Hospital, University of Montreal, Montreal, QC, Canada; 8 Centre for Interdisciplinary Research in Rehabilitation, Montreal, QC, Canada; 9 Institute of Public Health Research of the University of Montreal, Montreal, QC, Canada; 10 Orthopaedics Research Laboratory, Research Center, Sacré-Coeur Hospital, University of Montreal, Montreal, QC, Canada; 11 Department of Surgery, Faculty of Medicine, University of Montreal, Montreal, QC, Canada

**Keywords:** Physiotherapist, Healthcare service research, Musculoskeletal diseases and professional autonomy

## Abstract

**Background:**

In Canada, new models of orthopaedic care involving advanced practice physiotherapists (APP) are being implemented. In these new models, aimed at improving the efficiency of care for patients with musculoskeletal disorders, APPs diagnose, triage and conservatively treat patients. Formal validation of the efficiency and appropriateness of these emerging models is scarce. The purpose of this study is to assess the diagnostic agreement of an APP compared to orthopaedic surgeons as well as to assess treatment concordance, healthcare resource use, and patient satisfaction in this new model.

**Methods:**

120 patients presenting for an initial consult for hip or knee complaints in an outpatient orthopaedic hospital clinic in Montreal, Canada, were independently assessed by an APP and by one of three participating orthopaedic surgeons. Each health care provider independently diagnosed the patients and provided triage recommendations (conservative or surgical management). Proportion of raw agreement and Cohen’s kappa were used to assess inter-rater agreement for diagnosis, triage, treatment recommendations and imaging tests ordered. Chi-Square tests were done in order to compare the type of conservative treatment recommendations made by the APP and the surgeons and Student t-tests to compare patient satisfaction between the two types of care.

**Results:**

The majority of patients assessed were female (54%), mean age was 54.1 years and 91% consulted for a knee complaint. The raw agreement proportion for diagnosis was 88% and diagnostic inter-rater agreement was very high (κ=0.86; 95% CI: 0.80-0.93). The triage recommendations (conservative or surgical management) raw agreement proportion was found to be 88% and inter-rater agreement for triage recommendation was high (κ=0.77; 95% CI: 0.65-0.88). No differences were found between providers with respect to imaging tests ordered (p≥0.05). In terms of conservative treatment recommendations made, the APP gave significantly more education and prescribed more NSAIDs, joint injections, exercises and supervised physiotherapy (p<0.05). Patient satisfaction was significantly higher for APP care than for the surgeons care (p<0.05).

**Conclusion:**

The diagnoses and triage recommendations for patients with hip and knee disorders made by the APP were similar to the orthopaedic surgeons. These results provide evidence supporting the APP model for orthopaedic care.

## Background

Timely access to orthopaedic care is an important problem for the Canadian health care system. Presently, wait times to see an orthopaedic surgeon can exceed two years and with the rapidly aging population as well as the increased incidence of obesity, the need for orthopaedic care is expected to drastically increase in the coming years [1,2]. Efforts have been made to ensure timely access to orthopaedic care for the population yet, despite an investment in resources, wait times for a consult or for surgery remain excessively long [3-5].

Traditionally, the orthopaedic surgeon is the only health care provider seen when a patient is referred for an orthopaedic consultation but, with the increased demand for orthopaedic care, this model may no longer be viable. The orthopaedic surgeon is a highly trained surgeon and, with the current model in place, he/she spends a considerable amount of time seeing non-surgical cases that were referred to the orthopaedics service. In fact, certain studies have reported that 55% to 90% of patients newly referred to the orthopaedics department are not candidates for surgeries and would likely benefit from conservative treatment [6-9].

New models of care have emerged where interdisciplinary collaboration is favored; in these new models, physiotherapists replace orthopaedic surgeons as the first person seen when the patient is referred to the orthopaedics service and only surgical candidates or complex cases are referred to the surgeon. These models aim at improving access to care, with equal or better effectiveness, while containing costs and retaining patient satisfaction [10]. Many countries have already implemented these models and have defined an “advanced practice” or extended scope role for physiotherapists in which they formulate a diagnosis, triage potential surgical candidates, order imaging or laboratory tests and prescribe medication for patients with musculoskeletal disorders [11]. With the emergence of these new advanced practice models, there is a need to evaluate their efficacy and efficiency.

A recent systematic review done by our team concluded that there is emerging evidence suggesting that physiotherapists in advanced practice physiotherapy roles provide comparable or better care than physicians for patients with musculoskeletal disorders. However, these studies varied in terms of design and settings and often suffered from methodological biases [10]. This review also highlighted that validation of the APP model needs to include the following outcomes: 1- medical diagnostic agreement, triaging agreement of potential surgical candidates or clinical recommendations between advanced practice physiotherapists (APPs) and physicians; 2- effectiveness and efficiency of treatment provided by APPs; 3) economic evaluations (cost-effectiveness) of treatments provided by APPs; 4) patients satisfaction and 5) accessibility to care [10].

A few studies have looked at validation of the advanced practice physiotherapy model in the context of orthopaedic care. Daker-White *et al*. designed a randomized clinical trial (RCT) in a British orthopaedic clinic, comparing APP care to orthopaedic surgeons in training and found that APPs were as effective as junior doctors to treat patients with musculoskeletal disorders while ordering less diagnostic tests and reducing direct medical costs [12]. Patient satisfaction was also higher for the APP care. Other studies have looked at diagnostic validity and diagnostic concordance between APPs and surgeons for patients seen in an outpatient orthopaedic clinic and have found that for diagnosis and for determining whether patients would benefit from conservative care or surgical review, agreement may range from good to excellent (range κ = 0.69 to 1.00) [1,8,13] and treatment recommendations agreement is fair to very good (range κ = 0.52 to 0.70) [8,13]. In terms of patients’ satisfaction, one study found that patient satisfaction with advanced practice physiotherapy care was as high as with traditional care by orthopaedic surgeons for patients following total hip or knee replacement [14].

As part of the validation process following the recent implementation of an APP in the outpatient orthopaedic clinic at the Sacré-Coeur Hospital of Montreal, in Montreal, Canada, the objectives of the current study were to assess the diagnostic agreement of an APP compared to orthopaedic surgeons as well as to assess treatment concordance, health care resource use and patient satisfaction in this new model of care.

## Methods

### Settings

Consecutive patients were recruited from 02–2013 to 06–2013, from the waiting lists of the department of orthopaedic surgery at the HSCM, a supra regional university hospital with a tertiary trauma center.

### APP model at the HSCM outpatient orthopaedic clinic

The APP at the clinic had been in training (3 days/week) for the advanced practice role for 11 months. The APP’s initial background included 30 years of experience in sports and orthopaedic physical therapy and the APP was already trained to deal with direct access which is granted to all physiotherapists in the private sector of the province of Quebec. The physiotherapist’s training at the HSCM clinic was done in a residency type training program where the physiotherapist was taught by the three participating surgeons how to make diagnoses in the context of an orthopaedic clinic and how to review indications on ordering imaging tests. The initial implementation of this model did not include the independent prescription of medication or joint injections but the APP received some training, although not systematically, regarding these acts as they could potentially be added in the future to the APP’s role.

### Participants

Eligibility criteria for patients included: 1- aged 18 years or older; 2- referred for a new orthopaedic consult to one of the three participating orthopaedic surgeons; 3- referred for a knee or hip complaint; 4- resident of the province of Quebec and a beneficiary of the provincial universal health insurance coverage (Régie de l’Assurance Maladie du Québec - RAMQ); 5- able to understand and speak French; 6- able to legally consent to participate. Patients who had been previously treated by one of the three participating orthopaedic surgeons were excluded, as were patients who had undergone lower limb surgery in the past six months or who presented with more than two lower limb pathologies in addition to the one for which they were consulting.

### Data collection

Prior to being seen by the physiotherapist or the orthopaedic surgeon, patients completed a questionnaire where they provided anthropometric data as well as information on their education, employment, household income, household living status, and information on clinical variables such as the joint that was affected, the reason for consulting, the duration of their symptoms, the use of a walking aid and the presence of any comorbidities. Patients also completed the Lower Extremity Functional Scale (LEFS) questionnaire, which is used to evaluate the functional disabilities of a patient with a lower extremity disorder. The total is on 80 with a higher score indicating a higher functional status. The use of the LEFS in research studies has been validated and the LEFS is a reliable tool for assessing lower extremity functional status [15].

After independently seeing the patient, the orthopaedic surgeon and the physiotherapist each completed a form where they indicated their primary diagnosis and a secondary diagnosis if necessary. The clinical evaluation and test used by both providers to reach a diagnosis was not standardized and they could use any evaluation techniques or physical tests they felt necessary. The order of the evaluations by both providers varied, but it was not systematically randomized. The two health care providers had access to the patient’s medical file and any imaging tests previously ordered at the hospital. This was done to reflect the usual clinical evaluation of new patients where providers have access to this information. The health care providers were asked to record if they wanted to order any new imaging tests, and to specify the type of test. Finally, the orthopaedic surgeon and the APP were required to pick one of three options (conservative, surgical or undecided) with respect to the treatment approach for each patient and were to indicate whether or not they wished to make certain conservative treatment recommendations among a list of 9 conservative treatment options. These options were: 1) advice and education, 2) non prescription analgesics, 3) non-steroidal anti-inflammatories, 4) other medications, 5) joint infiltrations, 6) walking aids, 7) orthosis, 8) supervised physiotherapy, and 9) home exercises. The time in minutes it took for each health care practitioner to see the patient was also recorded. The APP and orthopaedic surgeon were blinded to each other diagnoses, triage and treatment recommendations and patients were asked not reveal any information from their first assessment to the second provider. After having been seen by both the orthopaedic surgeon and the APP, patients completed a modified version of the 9-item Visit-Specific Satisfaction Questionnaire (VSQ-9) [14,16]. Patients were informed that both health care providers would not have access to their responses. Each question was scored from 1 to 5 (1=excellent, 5=poor) and the responses were then transformed to a 0–100 score, with *excellent* being 100 and *poor* being 0 [16]. Between being seen by the physiotherapist and the orthopaedic surgeon, the patients also filled out a form where they were asked if their pain level increased after the first evaluation and in the event that it did, they were asked to indicate by how much it increased by picking one of the following three options: 1- pain was a bit stronger; 2- pain was moderately stronger; 3- pain was much stronger. To ensure that patients’ conditions were similar for both evaluations, patients were withdrawn from the study if their pain was either moderately or much stronger after the first evaluation.

### Analyses

Descriptive statistics were used to summarize the subjects’ characteristics. We calculated proportions of agreement as well as Cohen’s Kappa (with 95% confidence intervals) for diagnoses, imaging tests ordered, treatment approach recommendations and conservative treatment recommendations. Diagnostic concordance between the orthopaedic surgeon and the APP was evaluated by two independent reviewers in the following manner: the two independent reviewers classified the diagnoses made by the two health care practitioners using six categories for the knee (1- osteoarthritis; 2- ligament tear/rupture; 3- meniscal injury; 4- patellofemoral syndrome -PFS; 5- other; 6- undetermined) and five categories for the hip (1- osteoarthritis; 2- hip impingement syndrome; 3- tendonitis/bursitis; 4- other; 5- undetermined). The particular structure identified and the severity of the disorders were also considered in establishing concordance. The diagnosis of the orthopaedic surgeon was considered as the reference (gold) standard. The initial inter-rater agreement between the two independent reviewers for judging providers concordance was high (κ= 0.70; 95% CI: 0.47 – 0.87). Differences between the two independent reviewers were resolved by consensus. In the event where the APP and the orthopaedic surgeon disagreed on the primary diagnosis, the secondary diagnoses was taken into account to further evaluate diagnostic concordance.

For selected common knee disorders, such as osteoarthritis, ACL tear, meniscal injury, and patellofemoral syndrome, sensitivity, specificity, positive predictive value, negative predictive value, positive and negative likelihood ratios were calculated in order to determine diagnostic validity. The orthopaedic surgeons’ diagnosis was used as the reference standard. Asymptotic 95% CI were calculated.

The number of imaging tests ordered by the orthopaedic surgeons and the APP was compared using Chi-squared tests, as was the number of conservative treatment recommendations. Student t-tests were used to compare consultation duration measured in minutes for each class of providers as well as to compare patient satisfaction scores. For these two dependent variables, data were checked for normality.

Considering a potential raw agreement proportion of 80% between the two types of health care providers and a relative error of 20%, a sample size of 39 subjects was needed by pair of raters (orthopaedic surgeon and APP). Since three orthopaedic surgeon were participating in the study, we aimed at recruiting a sample size of 120 subjects [17]. Alpha level was set at 0.05. All statistical analyses were performed using the SPSS software version 20.

### Ethics

All patients signed a consent form prior to participating in the study. The study was approved by the Research Ethics Board of the Sacré Coeur of Montreal Hospital in Montreal, Canada.

## Results

### Participants

**Figure 1:**
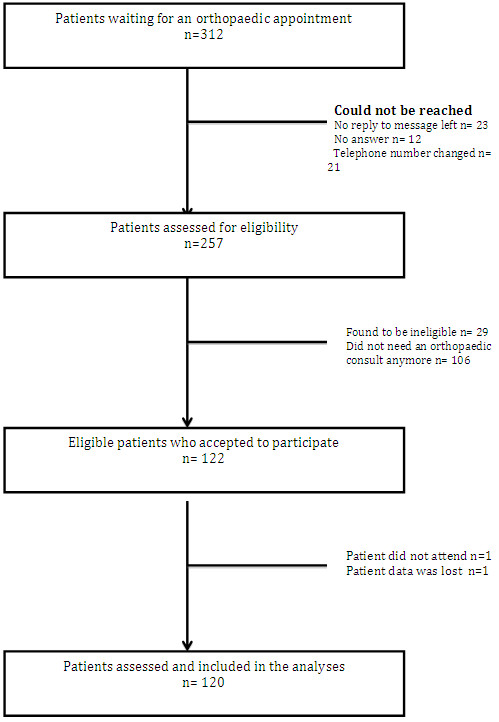
Flowchart of study participants.

Figure 1 presents a flow chart depicting recruitment of patient for the study. We identified 312 patients from the waiting lists of the three orthopaedic surgeons. Patients were contacted by telephone for initial participation. Fifty-six patients could not be reached; 106 patients did not need an appointment with an orthopaedic surgeon anymore and 29 were found to be ineligible because they did not meet the inclusion criteria. No patients refused to participate. This left us with 122 patients who were enrolled in the study and 120 patients whose data were used for analysis since one patient did not attend his appointment and data from one other patient was misplaced. The blinding of the practitioners was not broken during the study. No patients revealed information to the second provider following the evaluation of the first provider.

### Subjects’ characteristics

Table 1 presents selected characteristics of the participants. Subjects had a mean age of 54.1 years (SD: 15.9) and the majority was composed of women (54%). The majority of patients (56%) cited pain as the reason for consult with the orthopaedic surgeon and most of the patients (91%) consulted for a knee disorder. The mean Lower Extremity Functional Scale (LEFS) score of patients was 46.3 (SD: 19.1). 109 patients had imaging tests available in their file at the time of consult. Out of the 120 patients, only one patient indicated a higher pain level between the two sets of evaluations; the patient reported that the pain was a bit stronger but agreed to continue with the next evaluation and was therefore kept into the research protocol.

**Table 1:** Characteristics of the study participants (n=120)

**Characteristics**	**n (%)**	**Mean (SD)**
Age (years) ^‡^		54.1 (15.9)
Gender		
Female	65 (54)	
Male	55 (46)	
Body mass index (kg/m^2^) ^‡^		29.0 (6.1)
Living situation^§^		
Alone	17 (14.5)	
Education		
High school and less	45 (37.5)	
College and university	75 (62.5)	
Employment		
Employed	76 (63.3)	
Unemployed	10 (8.3)	
Retired	34 (28.3)	
Household income^¶^		
0 – 29 999$	45 (40.5)	
30 000 – 59 999$	23 (20.7)	
60 000$ +	43 (38.7)	
Joint affected		
Hip	11 (9)	
Knee	109 (91)	
Patient self reported reason for consult		
Pain	65 (56)	
Lack of joint mobility or joint instability	6 (5)	
Trauma	5 (4)	
Knee problem	31 (27)	
Hip problem	4 (3)	
Other	5 (4)	
Duration of symptoms (months)^╤^		59.0 (86.6)
Patients using walking aid	23 (19)	
Number of co-morbidities per patient		0.78 (0.95)
Patients with imagining diagnostic test results at time of consult	109 (91)	
Lower Extremity Functional Scale – LEFS score (%)^†^		46.3 (19.1)

SD= standard deviation.

^**‡**^n= 119.

^§^n= 117.

^¶^n= 111.

^╤^n= 106.

^**†**^n= 116.

### Diagnostic concordance between orthopaedic surgeons and APP

The overall proportion of agreement for the primary diagnosis between the orthopaedic surgeons and the physiotherapist was 88% and proportions for knee and hip primary diagnoses were 89% and 82% respectively. When the secondary diagnosis was taken into account, overall proportion of agreement increased to 93%; proportions for knee and hip diagnoses increased to 93% and 91% respectively. Overall, primary diagnostic inter-rater agreement was very high (κ=0.86; 95% CI: 0.80-0.93). For selected common knee disorders, proportion of agreement was highest for ACL tear diagnoses (100%) and lowest for PFS (86%). For knee disorders, diagnostic inter-rater agreement was very high (κ=0.87; 95% CI: 0.79-0.94). Cohen’s kappa could not be calculated because of high concordance and small number of hip cases (Table 2).

**Table 2:** Concordance between the advanced practice physiotherapist and orthopaedic surgeons on knee and hip primary diagnoses (n=120)

	**Raw agreement proportion (%)**		**Cohen’s kappa**	**95% CI**
Overall	106/120	(88)	0.86	0.80 – 0.93
Hip^†^	9/11	(82)		
Knee^‡^	97/109	(89)	0.87	0.79 – 0.94
Osteoarthritis	41/43	(95)		
ACL tear	12/12	(100)		
Meniscal injury	18/20	(90)		
Patellofemoral syndrome	12/14	(86)		

^**†**^ Cohen’s kappa could not be calculated because of high concordance and small number of hip cases (n=11).

^**‡**^ For knee disorders, raw agreement proportions (%) for selected common knee pathologies are also presented.

APP diagnostic validity compared to the diagnosis of the orthopaedic surgeons for selected common knee disorders (osteoarthritis, ACL rupture, meniscal injury and PFS) was very high as all sensitivity and specificity scores were over 0.90, except for the PFS diagnostic sensitivity, which was 0.86. Additionally, positive likelihood ratios were very high as all scores were over 25, and negative likelihood ratios were very low as all scores were 0.15 or less (Table 3).

**Table 3:** Advanced practice physiotherapist diagnostic validity for selected knee disorders compared to the orthopaedic surgeons diagnosis (n=109)

	**Sn [95% CI]**	**Sp [95% CI]**	**PPV [95% CI]**	**NPV [95% CI]**	**LR+[95% CI]**	**LR-[95% CI]**
Osteoarthritis	0.95	0.99	0.98	0.97	62.9	0.05
n= 43^†^	[0.85-0.99]	[0.92-1.00]	[0.88-1.00]	[0.90-0.99]	[9.0-440.6]	[0.01-0.18]
ACL tear	0.96	1.00	0.96	1.00	188.5	0.04
n= 12^†^	[0.72-1.00]	[0.95-1.00]	[0.72-1.00]	[0.95-1.00]	[11.9-2998.1]	[0.003-0.59]
Meniscal injury	0.90	0.97	0.86	0.98	26.7	0.10
n=20^†^	[0.70-0.97]	[0.91-0.99]	[0.65-0.95]	[0.92-0.99]	[8.7-82.0]	[0.03-0.39]
PFS	0.86	0.97	0.8	0.98	27.1	0.15
n= 14^†^	[0.60-0.96]	[0.91-0.99]	[0.55-0.93]	[0.93-0.99]	[8.7-84.4]	[0.04-0.53]

^**†**^ As diagnosed by the orthopaedic surgeon.

ACL = anterior cruciate ligament, PFS=patellofemoral syndrome.

SN= sensitivity, SP=specificity, PPV= positive predictive value, NPV= negative predictive value, LR+ = positive likelihood ratio, LR- = negative likelihood ratio.

### Health resource utilization

There were no significant differences between the orthopaedic surgeons and the APP when ordering any type of imaging test (p≥0.05) (Table 4). The highest raw agreement proportion between the orthopaedic surgeons and the APP was for ordering a CT scan with contrast (96%) and the lowest was for ordering X-rays (75%). Overall, inter-rater agreement was good (κ=0.65; 95% CI: 0.52-0.79) but for X-rays prescription inter-rater agreement was only fair (κ=0.48; 95% CI: 0.33-0.64) (Table 4).

**Table 4:** Differences in proportion and concordance for imaging test ordered between the advanced practice physiotherapist and orthopaedic surgeons (n=120)

	**Tests ordered by MD (%)**	**Tests ordered by APP (%)**	**Chi-squared test**	**p value**	**Raw agreement proportion**	**Cohen’s kappa**	**95% CI**
Any type of imaging tests^†^	67/120 (56)	79/120 (65)	2.518	0.113	82%	0.65	0.52 – 0.79
X-rays	50/120	60/120	1.678	0.195	75%	0.48	0.33 – 0.64
	(42)	(50)					
MRI	15/120	19/120	0.548	0.459	92%	0.66	0.46 – 0.85
	(13)	(16)					
CT scan with contrast	20/120	24/120					
	(17)	(20)	0.445	0.505	96%	0.78	0.63 – 0.93

MD= orthopaedic surgeon, APP= advanced practice physiotherapist, MRI= Magnetic resonance imaging, CT= computed tomography.

^†^ More than one type of imaging tests may be ordered for each patient.

### Treatment approach and conservative management concordance between orthopaedic surgeons and APP

There were 37 cases (31%) that were deemed to be surgical by the orthopaedic surgeon. The treatment approach inter-rater agreement was high (κ= 0.77; CI: 0.65-0.88). Raw agreement proportion on all treatment approaches was high (88%) as were raw agreement proportions on recommendation of a conservative approach (96%) and on recommendation of a surgical approach (89%). Raw agreement proportion on cases where the professionals were undecided on whether the patient would benefit most from a surgical or a conservative treatment was low (30%) (Table 5).

**Table 5:** Treatment approach concordance between the advanced practice physiotherapist and orthopaedic surgeons (n=120)

	**Raw agreement proportion (%)**		**Cohen’s kappa**	**95% CI**
Treatment approach				
All cases	106/120	(88)	0.77	0.65 – 0.88
Conservative	70/73	(96)		
Surgical	33/37	(89)		
Undecided	3/10	(30)		

The APP gave more advice and education to the patient, prescribed non-steroidal anti-inflammatories or joint infiltrations more often and recommended supervised physiotherapy or home exercises more frequently than the orthopaedic surgeon (p<0.001) (Table 6).

**Table 6:** Concordance between the advanced practice physiotherapist and orthopaedic surgeons for conservative treatment recommendations (n=120)

	**MD approach (%)**	**APP approach (%)**	**Chi-squared test**	**p value**	**Raw agreement proportion**
Advice and education	81/120	117/120	37.403	<0.001*	65%
	(68)	(98)			
Non prescription analgesics	2/120	4/120	0.648	0.408	97%
	(2)	(3)			
Non-steroidal anti-inflammatories	29/120	56/120	13.280	<0.001*	68%
	(24)	(47)			
Other medications	5/120	5/120	0.000	0.626	95%
	(5)	(5)			
Joint infiltrations	13/120	51/120	30.767	<0.001*	68%
	(11)	(43)			
Walking aids	1/120	0/120	N/A	N/A	99%
	(0.8)	(0)			
Orthosis	14/120	21/120	1.639	0.200	91%
	(12)	(18)			
Supervised physiotherapy	19/120	74/120	53.105	<0.001*	53%
	(16)	(62)			
Home exercises	12/120	101/120	132.467	<0.001*	23%
	(10)	(84)			

MD= orthopaedic surgeon, APP= advanced practice physiotherapist.

*p<0.05.

### Consult time and patient satisfaction

The orthopaedic surgeon took significantly less time to see patients (11.2 minutes, SD:3.3) than the APP (13.0 minutes, SD:3.8) (p <0.001). Patient satisfaction was higher for the APP (93.2%, SD 13.5) than for the orthopaedic surgeons (86.1%, SD 23.3) (p<0.001) (Table 7).

**Table 7:** Comparison between visit time length and patient satisfaction for orthopaedic surgeons and the advanced practice physiotherapist (n=112)

	**Mean value for MD (SD)**		**Mean value for APP (SD)**		**Mean difference (SD)**		**95% CI**	**Student *t*-test**	**p value**
Visit time length	11.2	(3.3)	13.0	(3.8)	1.8	(4.2)	0.97- 2.7	4.2	<0.001*
(in minutes)^†^									
Patient satisfaction^‡^	86.1	(23.3)	93.2	(13.5)	7.1	(19.1)	3.5- 10.7	3.9	<0.001*

SD= standard deviation, MD= orthopaedic surgeon, APP= advanced practice physiotherapist.

^**†**^ n= 94.

^**‡**^ Satisfaction was measured using a modified visit-specific satisfaction instrument (VSQ-9) questionnaire (/100). A higher score signifies higher satisfaction.

*p<0.05.

## Discussion

The aim of this study was to assess the validity of the advanced practice physiotherapy model of care for patients with musculoskeletal disorders in an orthopaedic outpatient clinic.

It is important to demonstrate the validity of the APP models of care as these models are being implemented in different settings in Canada and elsewhere. These new models will possibly play a crucial role in increasing access to care for Canadians, especially for those suffering from musculoskeletal disorders, as broader implementation of these models is expected and different APP training programs are being created in many provinces to help develop a critical number of APPs.

For the APP model of care at the Sacré-Coeur Hospital of Montreal, we found very high concordance on diagnoses and on treatment approach between the APP and orthopaedic surgeons. Although not significant, the APP tended to order more imaging tests than surgeons. With respect to conservative treatment recommendations, the APP tended to make certain recommendations more often than the orthopaedic surgeon. On average, it took the APP slightly more time to see the patients than the orthopaedic surgeon but the patients reported being more satisfied with APP care. Overall, these findings support the APP model of care for patients in orthopaedic outpatient clinics.

For the APP model to be valid, it must be shown that the APP makes similar diagnoses as the orthopaedic surgeon. Our findings strongly support that APP can accurately diagnose and make treatment recommendations in the context of an outpatient orthopaedic clinic. Previous studies have also shown that APPs have similar diagnostic capabilities to orthopaedic surgeons [1,8,13] and when compared to other healthcare providers such as nurse practionners or family physicians the APPs’ diagnostic accuracy has been found to be significantly better [18]. The results of our study show high to very high diagnostic concordance between both providers, similar to the results of Aiken *et al*. who showed a 90% concordance between APPs and orthopaedic surgeons (κ= 0.69) when diagnosing knee and shoulder musculoskeletal impairments [13]. In our study, there was divergence between the APP and the surgeon in only 14/120 cases and when considering the secondary diagnosis, the number of divergent cases fell to 9/120 participants. It is important to point out that, in this study, the orthopaedic surgeon was considered the reference standard and we cannot exclude the possibility that the surgeon’s diagnosis may have been erroneous for some cases; therefore, the lack of agreement for the 9 cases might may not necessarily be the results of an erroneous diagnosis by the APP. The inclusion of another surgeon into the protocol to independently compare the diagnosis of the surgeons would have been able to partly address this problem, but because of feasibility issues this was not included in the present protocol. For a subgroup of patients (n=32) with appropriate imaging tests, we were able to use the diagnosis of the radiologist as a reference standard to compare both surgeons and the APP diagnostic ability. The agreements between the orthopaedic surgeon and radiologist and between the APP and the radiologist were in fact the same at 75% (result not shown), further suggesting that APP and surgeons have similar diagnostic capability. It is important to point out that to reflect usual clinical practice, providers, APP and surgeons, had access to the patient’s medical file and previous imaging tests. Although unlikely, as patient’s information in the medical file was often not related or incomplete and previous imaging tests were not appropriate or outdated, this could have enhanced the concordance between providers. In terms of specific diagnosis for common knee disorders (osteoarthritis, ACL tear, meniscal injury and PFS), the APP’s diagnostic ability also yields very high sensitivity and specificity as well as high likelihood ratios confirming his ability to correctly diagnose patients with common knee disorders.

A key aspect of this new model of care is the ability of the APP to adequately triage the surgical candidate (treatment approach recommendations: surgical or conservative approach) as only patients requiring surgery should be referred to the surgeons. We showed high concordance between the APP and surgeons for triage of possible surgical candidates, in agreement with other studies [1,8,19]. Interestingly, we found that only 31% were deemed surgical candidates by the surgeon supporting the notion that orthopaedic surgeons receive a large number of inappropriate referrals [6-8].

We did find differences between APP and orthopaedic surgeons with respect to provision of advice/education, and recommendations for supervised physiotherapy or home exercises, consistent with the findings of Ball *et al*. [20]. Although it remains to be investigated in the specific context of APPs treating orthopaedic patients, it can be argued that supervised physiotherapy and home exercises can be beneficial to the patient since early intervention may prevent chronic problems [21]. In our study, the physiotherapist tended to prescribe non-steroidal anti-inflammatories (NSAIDs) and joint infiltrations more often than the orthopaedic surgeons. We may attribute this difference between the two professionals to the fact that the APP did not receive formal systematic training regarding medication and injection prescription as part of his initial residency program; nonetheless, it was evaluated in this study. Another possibility is that, since the APP recommended physical therapy or exercises more frequently than the orthopaedic surgeon, the APP prescribed NSAIDs and injections more often in order to limit pain and inflammation flare up that sometimes happens with the initiation of a rehabilitation program. This is often the case with patients suffering from osteoarthritis and current practice guidelines recommend the use of NSAIDs, joint infiltration in conjunction with the implementation of an exercise program and physiotherapy [22]. Another explanation could be that for surgical candidates, surgeons felt that the only therapeutic approach required was surgery and while waiting for the intervention no medication was necessary. In fact, among the 37 participants deemed surgical candidates by the surgeons, only one (3%) was prescribed NSAIDs by the orthopaedic surgeons while among the patients not requiring surgery, 28 (34%) were prescribed NSAIDs (data not shown). Since it differs from standard physiotherapy practice, medication and injection prescription is certainly an area where physiotherapists need formal training to integrate adequately these treatment modalities and when done so, these new roles have been taken on successfully by APPs in various countries [11].

With respect to utilization of resources, it must be shown that APPs do not request more imaging tests than orthopaedic surgeons when making a diagnosis and managing a patient. Our study demonstrated that there were no significant differences between the number of tests ordered by the APP and the orthopaedic surgeons, although the APP tended to order slightly more imaging tests and for X-rays, the concordance between the APP and surgeons was only fair. We believe that with additional training and experience in the new advanced practice role, that this issue would resolve as other studies have shown that APPs do not order more x-rays or MRIs than orthopaedic surgeons [18,20].

The APP took more time to see the patient than the orthopaedic surgeons, however, the difference was very small. The slightly increased time the APP spent with the patient may be due to the additional advice and prescribed home exercises.

Lastly, when attempting to implement a new model of care, patient satisfaction must be taken into account. In our study, patients reported a higher satisfaction with the APP consultation than with the orthopaedic surgeon visit which is consistent with other studies [2,12]. The higher reported satisfaction may be due to the fact that the APP spent more time with the patient and gave more advice, education, and exercises.

### Strengths and limitations

One of the strengths of our study was that it included a relatively large sample size of 120 consecutive patients presenting with a variety of different orthopaedic condition that affected the knee. However, we only had 11 cases with hip problems. Unlike other studies that have been done on advanced practice physiotherapy, our study used a systematic approach with two independent raters to determine diagnostic agreement between the orthopaedic surgeons and the APP. Another strength of our study was that it compared the diagnostic accuracy of the APP to three physicians; however, it would have been optimal to also have more than one APP and more than one clinical setting in order to increase generalizability. This was not possible since APP is an emerging role and few individuals have the required training. As mentioned earlier, having a second orthopaedic surgeons to compare the initial orthopaedic surgeon’s diagnosis may have helped when evaluating the concordance with the APP. However, since for most dependent variables the concordance was high, we do not believe it would have changed our conclusions. Our study on the validity of the APP model of care is one of the few to simultaneously evaluate critical outcomes of the advanced practice physiotherapy model such as diagnostic and treatment approach concordance, use of healthcare resources and patient satisfaction. Although the highest level of evidence advocating for the advanced practice physiotherapy model would have come from a randomized controlled trial (RCT), it would be difficult to undertake at this stage of implementation of the advanced practice physiotherapy model, with so few individuals working as APPs. In the near future, with the expansion of the model, we do advocate the realization of RCTs. One other outcome that was not reported in this study is the effect of the new APP model of care on wait time to see the APP or the surgeon. Following full implementation of the APP in this new role at the orthopaedic outpatient clinic, the effects on wait time for a consult or for surgery will be monitored.

## Conclusion

We found very high concordance on diagnoses and on treatment approach between the APP and orthopaedic surgeons. Although not significant, the APP tended to order more imaging tests than surgeons. With respect to conservative treatment recommendations, the APP made certain recommendations more often than the orthopaedic surgeons. On average, it took the APP slightly more time to see the patients than the orthopaedic surgeon but the patients reported being more satisfied with APP care. Overall, these findings support the APP model of care for patients seen in orthopaedic outpatient clinic. Future work should focus on the best way to systematically educate APPs for the advanced practice physiotherapy role and on the best way to implement the APP model in outpatient orthopaedic clinics.

## Competing interests

The authors declare that they have no competing interests.

## Authors’ contributions

FD participated in the design, coordination, collection of data and drafted the manuscript. PT participated in the collection of data, performed the statistical analysis and drafted the manuscript. JSR participated in the design and helped draft the manuscript. LJW participated in the design and helped draft the manuscript. ML participated in the design, collection of data and helped draft the manuscript. ML participated in the design and helped draft the manuscript. SG participated in the collection of data and helped draft the manuscript. DEF participated in the design and helped draft the manuscript. JCF participated in the design, coordination, collection of data and helped draft the manuscript. All authors read and approved the final manuscript.

## Pre-publication history

The pre-publication history for this paper can be accessed here:

http://www.biomedcentral.com/1471-2474/14/162/prepub
